# Reduced Membrane CD163 Expression in 7-Oxysterol-Induced Apoptosis Accompanied by Elevated Oxidative Stress

**DOI:** 10.3390/cells15131170

**Published:** 2026-06-27

**Authors:** Wei Li, Xi-Ming Yuan

**Affiliations:** 1 Clinical Department of Obstetrics and Gynecology in Linköping, Region Östergötland, SE 581 85 Linköping, Sweden; 2 Department of Biomedical and Clinical Sciences, Linköping University, SE 581 85 Linköping, Sweden; 3 Occupational and Environmental Medicine in Linköping, Department of Health, Medicine and Caring Sciences, Linköping University, SE 581 85 Linköping, Sweden; ximing.yuan@liu.se

**Keywords:** apoptosis, atherosclerosis, CD163, macrophage, monocyte, oxysterols, ROS

## Abstract

**Highlights:**

**What are the main findings?**

- Exposure to 7-oxysterols induced a dose-dependent reduction in cell surface CD163 expression.
- CD163 expression was inversely correlated with both apoptotic cell death and oxidative stress levels.

**What is the implication of the main finding?**

- Cell surface CD163 may have a potential function in macrophage survival and plaque stability in atherosclerotic lesions.

**Abstract:**

CD163 is a transmembrane scavenger receptor predominantly expressed by activated M2-like macrophages and is involved in inflammatory processes. Oxysterols, which accumulate in atherosclerotic lesions, are known to induce oxidative stress and apoptosis in macrophages. However, the relationship between CD163 expression and apoptosis induced by oxysterols remains poorly understood. Our brief report presents an examination of the effects of an atheroma-relevant mixture of 7β-hydroxycholesterol and 7-ketocholesterol (2mix) on cell surface CD163. THP-1 monocytes/macrophages were exposed to 2mix, and the surface expressions of CD163, apoptosis, and reactive oxygen species (ROS) production were assessed using flow cytometry and fluorescence microscopy. Exposure to 7-oxysterols induced a dose-dependent reduction in cell surface CD163 expression, with significant decreases observed in R1 and R2 cell populations but not in R3. This decrease was accompanied by a significant increase in apoptosis and ROS production. Notably, CD163 expression was inversely correlated with both apoptotic cell death and oxidative stress levels. Our findings suggest that macrophage surface CD163 may exert a protective role against 7-oxysterol-induced apoptosis and oxidative stress. This indicates a potential function of CD163 in macrophage survival and highlights its possible importance for plaque stability in atherosclerotic lesions.

## 1. Introduction

Atherosclerotic cardiovascular diseases have an inflammatory pathogenesis in which macrophages play a significant role in lesion initiation and progression. During plaque development, macrophage turnover and various forms of cell death contribute to plaque instability and the risk of related clinical events [1]. Within atherosclerotic plaques, macrophages differentiate into distinct functional subtypes under the influence of the vascular microenvironment. These macrophages can subsequently undergo cell death due to iron exposure or lipid overload through mechanisms such as apoptosis, necrosis, and ferroptosis. The accumulation of cellular debris and oxysterol-related pro-inflammatory signals further accelerate plaque growth and destabilization [2,3].

CD163 is a transmembrane protein that is exclusively expressed on the surface of monocytes and tissue-resident macrophages. It is commonly associated with activated (M2-like) macrophages, which are involved in anti-inflammatory processes and tissue repair. However, CD163+ macrophages have also been linked to the progression of atherosclerotic plaques [4,5]. In human carotid atherosclerosis, an increased number of CD163+ macrophages are associated with plaque vulnerability, supporting the suggestion that these cells contribute to clinically significant events [4]. In mice, CD163-expressing macrophages act as a protective mechanism, mitigating the deleterious effects of TWEAK on atherosclerotic plaque development and progression [5]. The best-characterized function of CD163 is its role in clearing the cell-free hemoglobin released during hemolysis by macrophages. This process depends on the plasma protein haptoglobin, which binds hemoglobin, and the endocytic hemoglobin receptor HbSR/CD163. The HbSR/CD163 activity and related pathway may contribute to pathological processes, including foam cell formation and the apoptosis of macrophages within the vascular wall [6].

Exposure to oxidized low-density lipoprotein (oxLDL) has been shown to upregulate CD163 expression in macrophages, thereby linking altered lipid metabolism to inflammatory processes within atherosclerotic plaques [7,8]. Oxysterols represent the major cytotoxic components of oxLDL and exist in mixed forms within human atheroma lesions, being toxic to a variety of cell types in the vascular wall [9,10]. The ability of oxysterols to trigger cell death and activate oxidation and inflammation may contribute to the development of atherosclerotic cardiovascular diseases [11,12]. We previously showed that the atheroma-relevant oxysterols 7β-hydroxycholesterol (7beta) and 7-ketocholesterol (7keto), combined in lesion-mimicking mixtures, exert synergistic toxic effects that enhance cell death [13,14]. However, it remains unknown whether CD163 plays a role in the macrophage death induced by these atheroma-relevant oxysterols.

In this project, we aimed to examine whether exposure to 7-oxysterol in the form of 2mix affects cell surface CD163 expression in THP-1 monocytes/macrophages and whether changes in this expression influence ROS production and apoptosis induced by 7-oxysterol.

## 2. Materials and Methods

### 2.1. Cell Cultures and Experimental Conditions

The human monocytic cell line THP-1 was maintained in RPMI 1640 medium (Gibco, Paisley, UK) supplemented with 10% fetal bovine serum (FBS), 2 mM L-glutamine, 100 U/mL of penicillin G, and 100 µg/mL of streptomycin. Cells were cultured at 37 °C in a humidified incubator containing 5% CO_2_ and 95% air. For experiments, cells were seeded at a density of approximately 3 × 10^5^ cells/mL. THP-1 cells were exposed to a mixture of 7keto and 7beta (Sigma, St. Louis, MO, USA) in a 1.8:1 ratio (2mix) at final concentrations of 14, 28, or 56 µM for 24 h. 7beta and 7keto were dissolved in ethanol to prepare a stock solution with a concentration of 10 mM. Various concentrations of 7beta, 7keto, and 2mix were initially tested in different cell lines including THP-1 cells [13,15]. Ethanol (0.28%, *v*/*v*) as vehicle control was tested in our previous experiments and showed no cytotoxic effect. In some experiments, THP-1 cells were differentiated into macrophages in coverslips in 35 mm culture dishes by incubation with phorbol myristate acetate (300 nM) for 24 h. After washing with culture medium, the cells were further cultured for 48 h under standard culture conditions before initiating the experiments [15].

### 2.2. Immunocytochemistry

THP-1 control and treated cells were collected, immunostained for CD163, and analyzed by flow cytometry. The cells were fixed with 1% paraformaldehyde for 20 min, washed with PBS, and incubated without permeabilization with rabbit anti-human CD163 (1:50; Santa Cruz biotechnology, Dallas, TX, USA) at 22 °C for 1 h or rabbit anti-human ferritin (DAKO, Carpinteria, CA, USA) at 4 °C overnight [16]. After washing, the cells were incubated with goat anti-rabbit-FITC (1:200; Invitrogen, Carlsbad, CA, USA) at 22 °C for 1 h. CD163 or ferritin fluorescence intensity was determined by flow cytometry or fluorescence microscopy. To validate the staining protocol, additional experiments were performed using fixation with either 1% or 2% PFA, combined with permeabilization (0.1% saponin and 5% serum in PBS). No difference in CD163 expression was observed between control and 2mix-treated cells following permeabilization after 1% PFA fixation. By contrast, CD163 MFI increased among 2mix-treated cells after fixation with 2% PFA and permeabilization. Furthermore, isotype controls confirmed the specificity of staining, showing no detectable signal.

We previously showed that 2mix induces cell death involving apoptosis and post-apoptotic necrosis [13]. In the present study, we examined CD163 expressions in different cell populations following 2mix treatment. Cell size and granularity in the control and treated samples were analyzed by gating three morphologically distinct populations (R1, R2, R3) based on forward scatter (FCS-H) and side scatter (SSC-H). The gating was constantly initiated with the control samples, which were then used to gate the other samples. R1 represents the major population with normal cell size and granularity; R2 consists of the cell population with normal size and increased granularity; and R3 consists of the cell population with reduced size and low granularity. In some experiments, immune-stained cells were additionally stained with Hoechst 33342 (10 μg/mL; Sigma, St. Louis, MO, USA) and examined by fluorescence microscopy.

### 2.3. Intracellular ROS

Intracellular reactive oxygen species (ROS) were quantified by flow cytometry using dihyroethidine (DHE; Molecular Probes, Eugene, OR, USA) staining, which is a fluorescent technique used to detect intracellular ROS, specifically superoxide radicals in living cells [17]. Briefly, cells from the respective experimental groups were harvested and incubated with 10 μM DHE for 15 min at 37 °C in the dark. Following staining, the cells were analyzed with flow cytometry and fluorescence microscopy. DHE median fluorescence intensity (MFI) was documented and used to quantify intracellular ROS.

### 2.4. Cell Viability and Apoptosis

The morphology and viability of control and 2mix-treated cells were first assessed by light microscopy. Apoptosis was initially assayed by morphological assessment, either directly in living cells using phase-contrast light microscopy or in cells fixed with 4% formaldehyde and stained according to Wright–Giemsa. Cells exhibiting shrinkage or pyknotic or fragmented nuclei were considered apoptotic [13]. To further quantify apoptosis, phosphatidylserine externalization was analyzed using flow cytometry following Annexin V/propidium iodide (PI) staining. Briefly, control and treated cells were harvested, washed with PBS, and incubated on ice for 10 min with Annexin V-FLOUS (Roche, Penzberg, Germany) and PI. The stained cells were then immediately analyzed by flow cytometry. Cells were classified as viable (Annexin V-/PI-), early apoptotic (Annexin V+/PI-), late/post-apoptotic necrotic (Annexin V+/PI+), or necrotic (Annexin V-/PI+). Nuclear condensation and fragmentation were also identified by florescence microscopy after Hoechst 33342 staining.

### 2.5. Statistics

For statistical analysis, a one-way ANOVA was followed by the Newman–Keuls post hoc test. The results are presented as means ± SEM. Correlations were assessed using the nonparametric Spearman correlation coefficient, and a *p*-value ≤ 0.05 was considered statistically significant.

## 3. Results

### 3.1. Exposure to 7-Oxysterols Induces a Dose-Dependent Reduction in Cell Membrane CD163 Expression

We first examined the cell surface expression of CD163 in THP-1 monocytes following exposure to the 7-oxysterol mixture (2mix). Because 2mix is cytotoxic and induces both apoptosis and post-apoptotic necrosis, we began by evaluating cell morphology using flow cytometry, specifically analyzing forward scatter (FSC-H; cell size) and side scatter (SSC-H; granularity) in CD163-stained cells.

**Figure 1:**
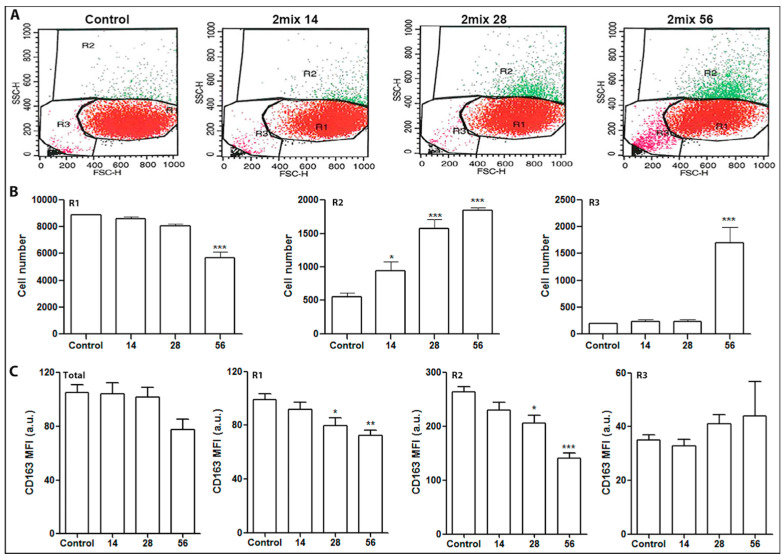
Treatment with 2mix causes a cell population shift and is linked to a reduction in cell surface CD163 expression. THP-1 monocytes were exposed to increasing concentrations of 2mix (14, 28, or 56 µM) for 24 h, stained for surface CD163, and analyzed by flow cytometry. (**A**) Representative flow cytometry plots showing cell populations in the control and after treatment with different 2mix concentrations. R1 represents cells with normal size and granularity; R2 represents cells with normal size but increased granularity; and R3 represents cells with reduced size and low granularity. (**B**) The quantification of the cell numbers counted within the R1, R2, and R3 populations. (**C**) The dose-dependent reduction in CD163 expression induced by 2mix in the R1 and R2 populations but not in R3. Data in (**B**,**C**) are means ± SEM from three experiments performed in duplicates; * *p* < 0.05, ** *p* < 0.01, and *** *p* < 0.001 vs. controls.

Compared with control cells, 2mix induced a dose-dependent decrease in cell size and an increase in granularity (Figure 1). A representative flow cytometry dot plot in the control and different concentrations of 2mix-treated cells is shown in Figure 1A. As shown in Figure 1B, exposure to 2mix resulted in a dose-dependent shift from the R1 to the R2 population, which is likely to reflect early apoptotic changes. By contrast, an increase in the R3 population was only observed at the highest concentration of 2mix (56 μM), indicating the presence of cells with reduced membrane integrity consistent with post-apoptotic necrosis.

We next analyzed CD163 immunointensity across different cell populations and found that exposure to 2mix induced a dose-dependent decrease in cell membrane-associated CD163 in both the R1 and R2 populations (Figure 1C). Interestingly, a slight increase rather than a decrease in the CD163 signal was observed in the R3 population following exposure to 56 μM 2mix. This increase likely reflects intracellular antibody penetration resulting from compromised membrane integrity, leading to elevated intracellular CD163 fluorescence (Figure 1C).

**Figure 2:**
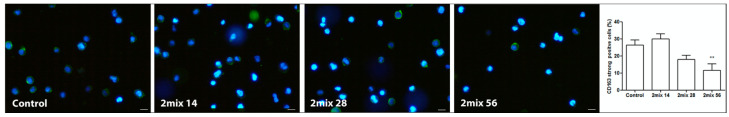
Treatment with 2mix reduces cell surface expression of CD163. THP-1 monocytes were treated with increasing concentrations of 2mix for 24 h; fixed with 1% PFA, without permeabilization; immune-stained for CD163; and assayed by fluorescence microscopy. Representative fluorescence micrographs of THP-1 control cells and cells treated with different concentrations of 2mix (14, 28, or 56 μM, bars = 50 µm. Quantification of percentage of THP-1 monocytes displaying strong surface CD163 expression (bar graphs in right panel). Data represents means ± SEM from 18 photographs in each group from three independent experiments performed in duplicates; ** *p* < 0.01 vs. controls.

The reduction in membrane CD163 after 2mix exposure was further confirmed by fluorescence microscopy (Figure 2).

**Figure 3:**
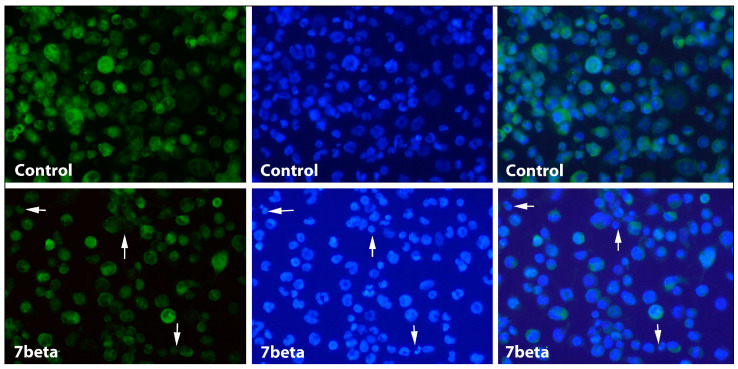

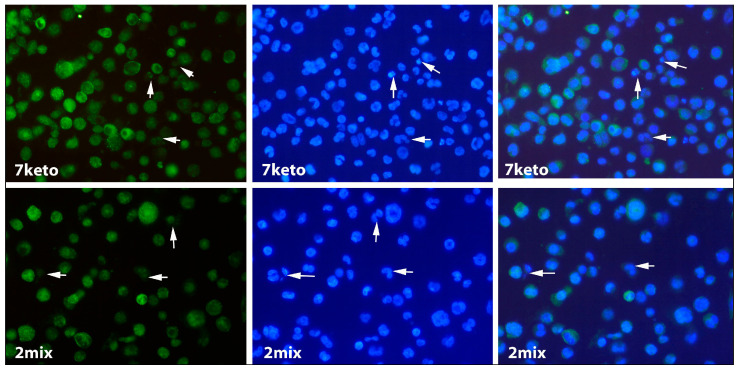
Reduced CD163 expression in THP-1 macrophages following treatment with 7beta, 7keto, or their combination (2mix). THP-1 macrophages cultured on coverslips in 35 mm dishes were treated for 24 h with 28 μM 7beta, 7keto, or 2mix. Cells were subsequently fixed with 1% PFA (without permeabilization), immune-stained for CD163, and analyzed by fluorescence microscopy. CD163 staining (green fluorescence, left panels) and nuclear staining (blue fluorescence, middle panels) were acquired separately. The right panels show the corresponding merged (overlay) images. Note that apoptotic cells displaying nuclear condensation and fragmentation exhibited minimal to no CD163 expression at the cell membrane (arrows).

In some experiments, we assessed CD163 immunostaining in THP-1 macrophages treated with 7beta, 7keto, or their combination (2mix) (Figure 3). The reduction in CD163 expression was observed in 2mix-, 7beta-, and 7keto-treated cells, especially in cells with nuclear condensation and fragmentation.

### 3.2. 7-Oxysterol Induces Dose-Dependent Apoptosis and Elevates ROS Production, and Both Are Inversely Associated with CD163 Expression

**Figure 4:**
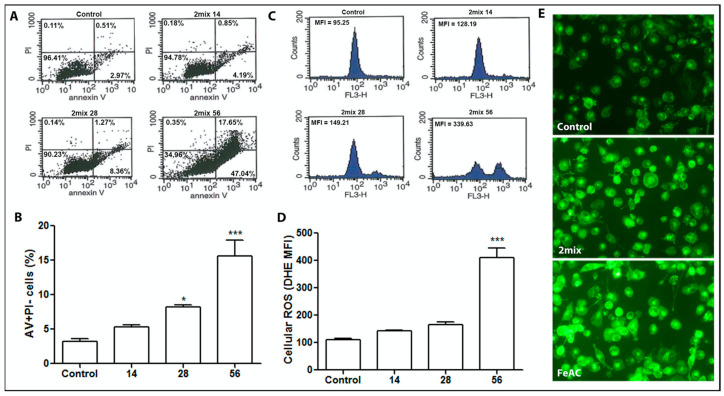
Treatment with 2mix induces dose-dependent apoptosis and increases cellular ROS and ferritin. (**A**–**D**) THP-1 monocytes were treated with increasing concentrations of 2mix for 24 h, stained with either AV/PI (**A**,**B**) or DHE (**C**,**D**), and assessed by flow cytometry. (**A**) Representative flow cytometry dot plots of AV/PI staining. Cells were classified as viable (AV-/PI-, lower left squares), early apoptotic (AV+/PI, lower right squares), and late apoptotic (AV+/PI+, upper right squares). (**B**) Percentage of cells with AV+/PI-. Data are means ± SE of three independent experiments performed in duplicate. * *p* < 0.05, *** *p* < 0.0001 vs. control. (**C**) Representative DHE fluorescence histograms showing increase in mean fluorescence intensity (MFI), indicating elevated intracellular ROS. (**D**) Quantification of DHE MFI. Data is presented as means ± SEM of three independent experiments performed in duplicate. *** *p* < 0.0001 vs. control. (**E**) Intracellular ferritin induction in THP-1 macrophages by exposure to 2mix as compared to ferric ammonium citrate-treated cells (50 μg/mL for 24 h).

We next investigated apoptosis and intracellular ROS formation following exposure to 2mix, as well as their relationship with CD163 expression. Treatment with 2mix induced a clear dose-dependent increase in apoptosis, as determined by flow cytometry using AV/PI staining (Figure 4A,B) and corresponding to an increase in intracellular ROS levels (Figure 4C,D). This process was accompanied by a clear increase in ferritin expression induced by 2mix compared with iron-treated controls (Figure 4E).

**Figure 5:**
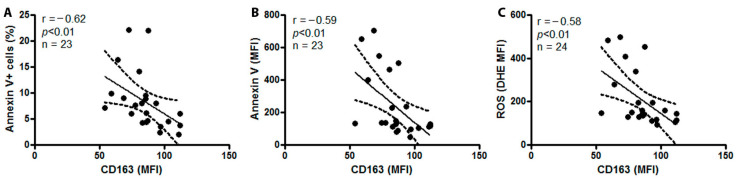
CD163 expression is inversely correlated with apoptosis and ROS production. THP-1 monocytes were treated and stained as described in Figure 1, Figure 2 and Figure 4, and the correlation between CD163 and apoptosis or ROS production was evaluated using the Spearman correlation test. (**A**,**B**) The correlation between CD163 MFI in the R1 population and apoptosis measured as the percentage of annexin V-positive cells (**A**) or annexin V MFI (**B**). (**C**) The correlation between CD163 MFI and ROS production. Data represent control cells and cells treated with different concentrations of 2mix from three independent experiments performed in duplicate.

To determine whether CD163 expression was correlated with apoptosis and ROS production, we performed Spearman correlation analyses comparing CD163 mean fluorescence intensity (MFI) with the frequency of Annexin V-positive cells, Annexin V MFI, and DHE MFI. CD163 expression showed a significant inverse correlation with apoptosis (Figure 5A,B) as well as with ROS production (Figure 5C). The CD163 MFI values shown in these figures were derived from the R1 population. The same inverse correlation was detected in the R2 population but not in the R3 population in dying cells.

## 4. Discussion

CD163^+^ macrophages have usually been considered atheroprotective due to their high expression of the anti-inflammatory cytokine IL-10 and heme-degrading enzyme hemeoxygenase, as well as their association with reduced oxidative stress [18,19]. These cells have also been involved in reversed cholesterol transport, thereby limiting foam cell formation [20]. However, the relationship between CD163^+^ macrophages and plaque stability in human atherosclerotic lesions remains unclear, as studies have reported conflicting results [4,5,21]. These discrepancies highlight the need for further investigations to clarify the functional roles of CD163 in the vascular biology of atherosclerosis.

Previous studies by our group [22] and others [23] have suggested that one reason that atherosclerotic lesions persist and continue to grow, rather than being efficiently cleared by macrophages, is that certain plaque-derived substances, particularly oxysterols, exert cytotoxic effects on these cells. Such toxicity may impair macrophage function and limit their ability to remove accumulating lipids and cellular debris. Our previous work demonstrated that the oxysterols present in human atheroma, in atheroma-relevant proportions, display synergistic and pro-apoptotic effects [13,14]. Our present findings demonstrate that exposure to atheroma-relevant 7-oxysterol mixtures markedly reduces cell surface CD163 expression. This reduction indicates the functional impairment of CD163^+^ cells, likely associated with the diminished anti-inflammatory capacity of CD163. To our knowledge, this is the first study to examine cell surface CD163 expression in the context of atherosclerosis. The results provide additional support for our hypothesis that atheroma lesions represent a “death zone” enriched with toxic components, including 7-oxysterols, which may impair macrophage function and survival, thereby hindering plaque clearance.

As a substantial component of oxidized LDL, 7-oxysterols are compelling contributors to vascular instability, given their pronounced pro-oxidative, pro-inflammatory, and pro-apoptotic properties [24,25]. In this study, mixtures of 7-oxysterols were found to alter the distribution of cell populations, accompanied by a dose-dependent reduction in CD163 expression in the R1 and R2 populations but not in late/post-apoptotic cells (R3). These changes in both cell population distribution and surface CD163 expression suggest that CD163 downregulation occurs prior to the execution phase of apoptosis, highlighting a potential role for CD163 in regulating macrophage fate during atherosclerosis.

The oxysterol-induced changes in cell populations may reflect dynamic cellular turnover during atherosclerotic processes within the vascular wall. The main population (R1) represents normal cells characterized by intermediate size and granularity, whereas the R2 population consists of cells with increased granularity, potentially indicative of early apoptotic changes [26]. Cells in the R3 population display reduced cell volume and condensed chromatin, consistent with post-apoptotic morphology [27]. Several mechanisms may explain the observed population shifts during interactions between oxysterols and macrophages. Increased granularity may reflect early apoptosis, which is associated with nuclear condensation and fragmentation, while cell size remains unchanged [26]. Additionally, the treatment of monocytes/macrophages with oxysterols has been reported to induce foam-cell-like characteristics, as demonstrated by Oil Red O staining [28]. These lipid-laden foam cells typically exhibit increased granularity. Consistently, previous studies have shown that foam cells derived from oxysterol-treated monocytes or macrophages display elevated granularity without significant changes in cell size [28,29], supporting the observations in the present study.

### Limitations

1. CD163 expression is inversely associated with apoptosis and ROS production, although the underlying mechanisms remain unclear. As a scavenger receptor, CD163 plays a key role in limiting oxidative stress and modulating inflammatory signaling [30]. In contrast, oxysterols such as 7-ketocholesterol are established inducers of macrophage apoptosis and ROS generation.
2. Our findings support the central role of ROS in regulating macrophage survival and suggest that oxysterols may influence vascular pathology through the modulation of CD163 expression. This is consistent with current evidence linking oxysterols to an altered macrophage phenotype, including reduced CD163^+^ polarization [31].
3. Future work should focus on defining the impact of specific oxysterols on CD163 surface expression and clarifying its functional role in macrophage fate using gain- and loss-of-function approaches. A further investigation of the interaction between CD163-dependent pathways and ROS-driven apoptosis will be important to better understand oxysterol-induced cytotoxicity in atherosclerosis.

## 5. Conclusions

In conclusion, cell surface CD163 may exert a protective role against 7-oxysterol-induced apoptosis and oxidative stress. This indicates a potential function of CD163 in macrophage survival and highlights its possible importance for plaque stability in atherosclerotic lesions. However, our brief report warrants further mechanistic studies to clarify the causal relationship between surface CD163 expression and macrophage fate in atherosclerotic plaque instability.

## Data Availability

The data supporting the conclusions of this article will be made available by the authors on reasonable request.

## References

[1] De Meyer G.R.Y., Zurek M., Puylaert P., Martinet W. (2024). Programmed death of macrophages in atherosclerosis: Mechanisms and therapeutic targets. Nat. Rev. Cardiol..

[2] Poli G., Biasi F., Leonarduzzi G. (2013). Oxysterols in the pathogenesis of major chronic diseases. Redox Biol..

[3] Vejux A., Abed-Vieillard D., Hajji K., Zarrouk A., Mackrill J.J., Ghosh S., Nury T., Yammine A., Zaibi M., Mihoubi W. (2020). 7-Ketocholesterol and 7β-hydroxycholesterol: In vitro and animal models used to characterize their activities and to identify molecules preventing their toxicity. Biochem. Pharmacol..

[4] Bengtsson E., Hultman K., Edsfeldt A., Persson A., Nitulescu M., Nilsson J., Gonçalves I., Björkbacka H. (2020). CD163+ macrophages are associated with a vulnerable plaque phenotype in human carotid plaques. Sci. Rep..

[5] Gutiérrez-Muñoz C., Méndez-Barbero N., Svendsen P., Sastre C., Fernández-Laso V., Quesada P., Egido J., Escolá-Gil J.C., Martín-Ventura J.L., Moestrup S.K. (2020). CD163 deficiency increases foam cell formation and plaque progression in atherosclerotic mice. FASEB J..

[6] Madsen M., Graversen J.H., Moestrup S.K. (2001). Haptoglobin and CD163: Captor and receptor gating hemoglobin to macrophage lysosomes. Redox Rep..

[7] Cai Y., Wen J., Ma S., Mai Z., Zhan Q., Wang Y., Zhang Y., Chen H., Li H., Wu W. (2021). Huang-Lian-Jie-Du Decoction Attenuates Atherosclerosis and Increases Plaque Stability in High-Fat Diet-Induced ApoE-/-Mice by Inhibiting M1 Macrophage Polarization and Promoting M2 Macrophage Polarization. Front. Physiol..

[8] Bisgaard L.S., Mogensen C.K., Rosendahl A., Cucak H., Nielsen L.B., Rasmussen S.E., Pedersen T.X. (2016). Bone marrow-derived and peritoneal macrophages have different inflammatory response to oxLDL and M1/M2 marker expression-implications for atherosclerosis research. Sci. Rep..

[9] Dias I.H., Petler N., Harihararajah K., Kremenska Y., Anderson A.M., O’Connor M.S. (2026). Emerging role of 7-Ketocholesterol and hydroxylated 7-Ketocholesterol in the pathophysiology of disease. J. Steroid Biochem. Mol. Biol..

[10] Nury T., Yammine A., Ghzaiel I., Sassi K., Zarrouk A., Brahmi F., Samadi M., Rup-Jacques S., Vervandier-Fasseur D., Pais de Barros J.P. (2021). Attenuation of 7-ketocholesterol- and 7β-hydroxycholesterol-induced oxiapoptophagy by nutrients, synthetic molecules and oils: Potential for the prevention of age-related diseases. Ageing Res. Rev..

[11] Lizard G., Monier S., Cordelet C., Gesquière L., Deckert V., Gueldry S., Lagrost L., Gambert P. (1999). Characterization and comparison of the mode of cell death, apoptosis versus necrosis, induced by 7beta-hydroxycholesterol and 7-ketocholesterol in the cells of the vascular wall. Arterioscler. Thromb. Vasc. Biol..

[12] Vejux A., Lizard G. (2009). Cytotoxic effects of oxysterols associated with human diseases: Induction of cell death (apoptosis and/or oncosis), oxidative and inflammatory activities, and phospholipidosis. Mol. Asp. Med..

[13] Larsson D.A., Baird S., Nyhalah J.D., Yuan X.M., Li W. (2006). Oxysterol mixtures, in atheroma-relevant proportions, display synergistic and proapoptotic effects. Free Radic. Biol. Med..

[14] Lizard G. (2006). Oxysterol mixtures, a promising approach to investigate the biological effects of oxysterols: A commentary on “Oxysterol mixtures, in atheroma-relevant proportions, display synergistic and proapoptotic effects” by Larsson, Baird, Diinga Nyhalah, Yuan, and Li. Free Radic. Biol. Med..

[15] Laskar A., Eilertsen J., Li W., Yuan X.M. (2013). SPION primes THP1 derived M2 macrophages towards M1-like macrophages. Biochem. Biophys. Res. Commun..

[16] Smit J.W., Meijer C.J., Decary F., Feltkamp-Vroom T.M. (1974). Paraformaldehyde fixation in immunofluorescence and immunoelectron microscopy. Preservation of tissue and cell surface membrane antigens. J. Immunol. Methods.

[17] Abubaker A.A., Vara D., Eggleston I., Canobbio I., Pula G. (2019). A novel flow cytometry assay using dihydroethidium as redox-sensitive probe reveals NADPH oxidase-dependent generation of superoxide anion in human platelets exposed to amyloid peptide β. Platelets.

[18] Boyle J.J., Harrington H.A., Piper E., Elderfield K., Stark J., Landis R.C., Haskard D.O. (2009). Coronary intraplaque hemorrhage evokes a novel atheroprotective macrophage phenotype. Am. J. Pathol..

[19] Philippidis P., Mason J.C., Evans B.J., Nadra I., Taylor K.M., Haskard D.O., Landis R.C. (2004). Hemoglobin scavenger receptor CD163 mediates interleukin-10 release and heme oxygenase-1 synthesis: Anti-inflammatory monocyte-macrophage responses in vitro, in resolving skin blisters in vivo, and after cardiopulmonary bypass surgery. Circ. Res..

[20] Finn A.V., Nakano M., Polavarapu R., Karmali V., Saeed O., Zhao X., Yazdani S., Otsuka F., Davis T., Habib A. (2012). Hemoglobin directs macrophage differentiation and prevents foam cell formation in human atherosclerotic plaques. J. Am. Coll. Cardiol..

[21] Yunoki K., Inoue T., Sugioka K., Nakagawa M., Inaba M., Wada S., Ohsawa M., Komatsu R., Itoh A., Haze K. (2013). Association between hemoglobin scavenger receptor and heme oxygenase-1-related anti-inflammatory mediators in human coronary stable and unstable plaques. Hum. Pathol..

[22] Li W., Ostblom M., Xu L.H., Hellsten A., Leanderson P., Liedberg B., Brunk U.T., Eaton J.W., Yuan X.M. (2006). Cytocidal effects of atheromatous plaque components: The death zone revisited. FASEB J..

[23] Hegyi L., Hardwick S.J., Siow R.C., Skepper J.N. (2001). Macrophage death and the role of apoptosis in human atherosclerosis. J. Hematotherapy Stem Cell Res..

[24] Gargiulo S., Gamba P., Testa G., Leonarduzzi G., Poli G. (2016). The role of oxysterols in vascular ageing. J. Physiol..

[25] Lemaire-Ewing S., Prunet C., Montange T., Vejux A., Berthier A., Bessède G., Corcos L., Gambert P., Néel D., Lizard G. (2005). Comparison of the cytotoxic, pro-oxidant and pro-inflammatory characteristics of different oxysterols. Cell Biol. Toxicol..

[26] Wlodkowic D., Telford W., Skommer J., Darzynkiewicz Z. (2011). Apoptosis and beyond: Cytometry in studies of programmed cell death. Methods Cell Biol..

[27] Darzynkiewicz Z., Juan G., Li X., Gorczyca W., Murakami T., Traganos F. (1997). Cytometry in cell necrobiology: Analysis of apoptosis and accidental cell death (necrosis). Cytometry.

[28] Hayden J.M., Brachova L., Higgins K., Obermiller L., Sevanian A., Khandrika S., Reaven P.D. (2002). Induction of monocyte differentiation and foam cell formation in vitro by 7-ketocholesterol. J. Lipid Res..

[29] Lee Y.H., Chen S.Y., Wiesner R.J., Huang Y.F. (2004). Simple flow cytometric method used to assess lipid accumulation in fat cells. J. Lipid Res..

[30] Moestrup S.K., Møller H.J. (2004). CD163: A regulated hemoglobin scavenger receptor with a role in the anti-inflammatory response. Ann. Med..

[31] Buttari B., Segoni L., Profumo E., D’Arcangelo D., Rossi S., Facchiano F., Businaro R., Iuliano L., Riganò R. (2013). 7-Oxo-cholesterol potentiates pro-inflammatory signaling in human M1 and M2 macrophages. Biochem. Pharmacol..

